# Intrauterine Herpes Simplex Virus Infection Presenting With Fetal Sinus Bradycardia and Pericardial Effusion: A Case Report

**DOI:** 10.7759/cureus.97281

**Published:** 2025-11-19

**Authors:** Kaoru Ikeda, Mitsuhiro Haga, Rika Noguchi, Sumiko Era, Hirotaka Ishido

**Affiliations:** 1 Department of Pediatrics, National Defense Medical College Hospital, Tokorozawa, JPN; 2 Department of Pediatrics, Saitama Medical Center, Saitama Medical University, Kawagoe, JPN; 3 Department of Obstetrics and Gynecology, Saitama Medical Center, Saitama Medical University, Kawagoe, JPN

**Keywords:** disseminated herpes simplex virus infection, fetal bradycardia, fetal echocardiography, intrauterine hsv infection, pericardial effusion (pe), viral-induced myocarditis

## Abstract

Cardiac complications have rarely been reported in intrauterine herpes simplex virus (HSV) infection. We report a case of myocarditis associated with intrauterine HSV infection. Our patient’s mother was a 28-year-old woman with no known history of HSV infection. Fetal ultrasound at 26 weeks of gestation revealed sinus bradycardia and pericardial effusion. Cesarean section was performed due to placental abruption at 28 weeks of gestation. A male newborn was born with extensive skin erosions, hypotension, and persistent bradycardia unresponsive to resuscitation. Despite intensive treatment, he died within 24 hours of life. Postmortem computed tomography revealed multiple calcifications in the heart wall, suggesting the existence of myocarditis. HSV-2 was confirmed in skin exudates by viral culture, and the diagnosis of disseminated HSV infection was made. Intrauterine HSV infection can be associated with cardiac complications. Clinicians should be aware of the possibility of HSV infection when encountering fetuses with the signs of myocarditis.

## Introduction

Neonatal herpes simplex virus (HSV) infection is a severe disease associated with high mortality [1]. Intrauterine infection accounts for only 5% of neonatal HSV infections, but it can lead to severe systemic symptoms [1]. The classic triad of intrauterine HSV infection includes skin, central nervous system, and eye lesions [1]; other commonly affected organs include the liver and adrenal glands [2]. However, due to its rarity, the clinical course of intrauterine HSV infection is still largely unknown, and prenatal diagnosis is a major challenge. We encountered a case that presented with sinus bradycardia and pericardial effusion in utero, which was diagnosed as disseminated HSV infection after birth.

## Case presentation

**Figure 1:**
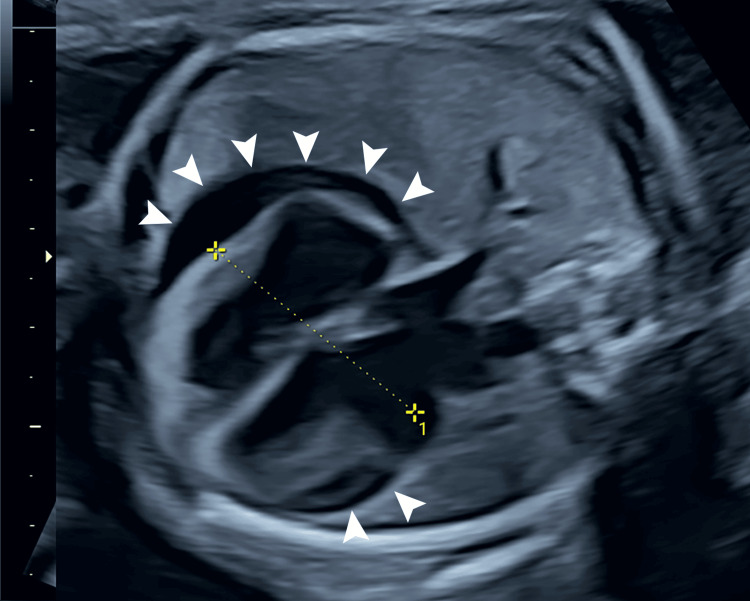
Fetal ultrasonography image at 26 weeks of gestation. The whole circumference of the heart was surrounded by an echo-free space of 7-8 mm (arrowheads), indicating pericardial effusion.

The patient’s mother was a 28-year-old, gravida 4 para 2, Asian woman with no known history of HSV infection. At 26 weeks of gestation, a fetal ultrasound showed sinus bradycardia (100/min) and a whole-circumference pericardial effusion measuring 7-8 mm in width (Figure 1).

The mother was admitted to our institution for observation; after admission, the fetus developed enlargement of the right lateral ventricle of the brain and multiple hyperechoic round lesions in the liver. At 28 weeks and three days of gestation, the mother suffered placental abruption, and an emergency cesarean section was performed.

**Figure 2:**
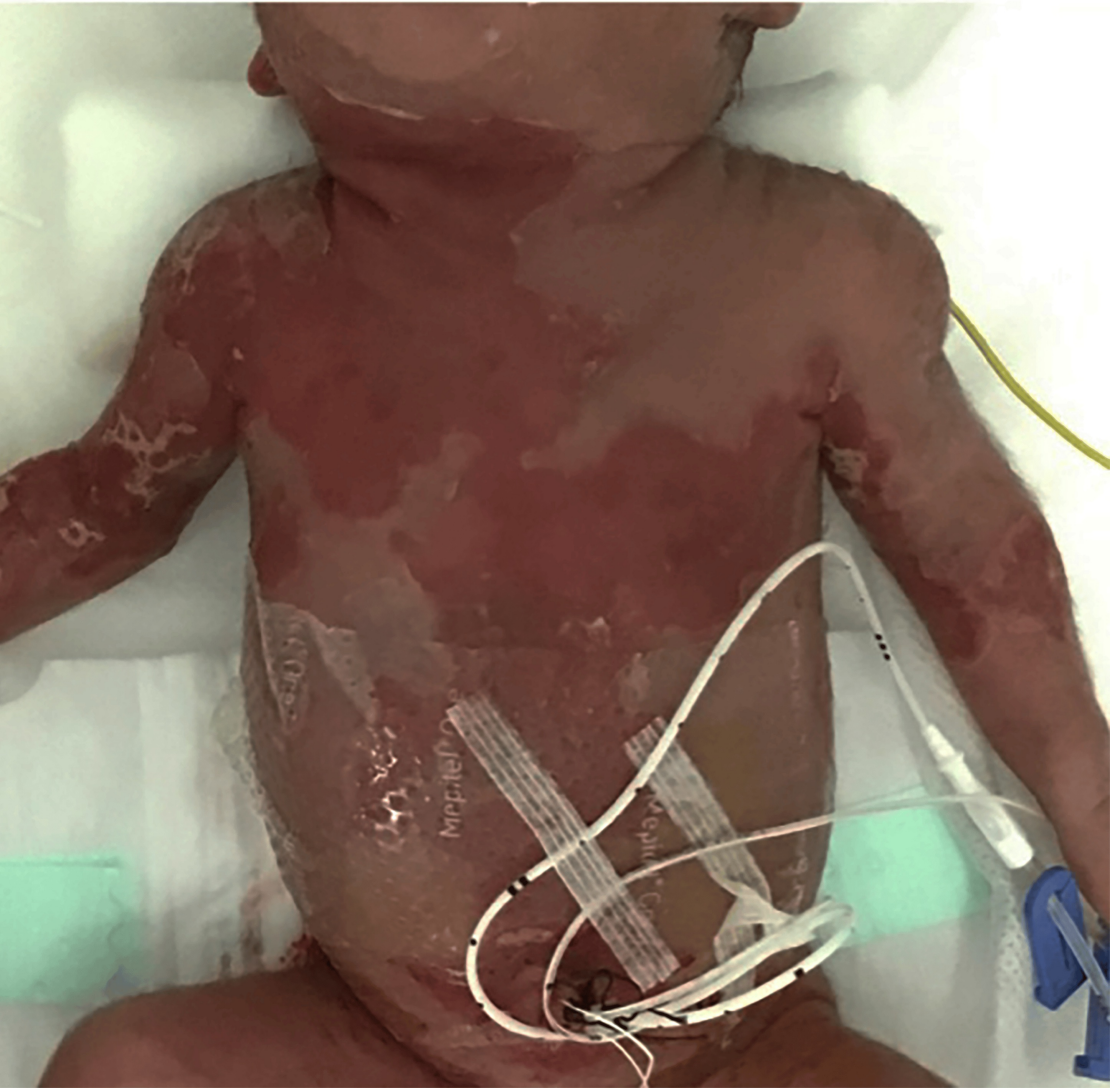
Skin findings at admission. Extensive skin erosions were observed all over the body.

The male neonate was born with a birth weight of 950 g. The Apgar scores were 4 at 1 min and 5 at 5 min. The skin was eroded all over the body at birth (Figure 2).

Bradycardia of around 60/min continued after birth. He was resuscitated with endotracheal intubation and intratracheal adrenaline administration, but bradycardia did not resolve after the procedures. He was admitted to our neonatal intensive care unit for further treatment. He suffered from severe respiratory insufficiency, requiring 100% FiO_2_ to maintain SpO_2_ above 90%. The heart rate was 60-70/min, and the mean arterial blood pressure was 24 mmHg. The laboratory data revealed anemia (hemoglobin 8.8 g/dL), low platelet count (2,600/μL), and severe coagulopathy (activated partial prothrombin time 63.3 sec.; prothrombin time-international normalized ratio 8.0; and fibrinogen <60 mg/dL). The echocardiography showed severe mitral and tricuspid regurgitation (Videos 1, 2).

**Video 1:** Tranthoracic echocardiography at admission (mitral regurgitation). Two-dimensional color Doppler echocardiography shows severe mitral regurgitation. Note that an echo-free space can be seen anterior to the right ventricle, indicating the existence of pericardial effusion.

**Video 2:** Transthoracic echocardiography at admission (tricuspid regurgitation). Two-dimensional color Doppler echocardiography shows severe tricuspid regurgitation.

**Figure 3:**
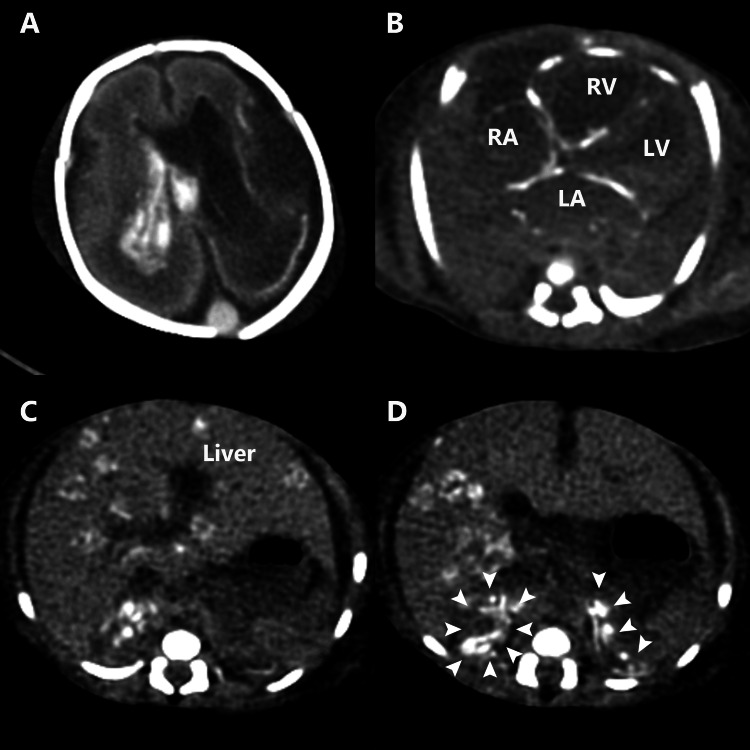
Postmortem computed tomography images. (A) Brain: the right lateral ventricle is dilated, and intraventricular hemorrhage is seen in the left lateral and third ventricles. (B) Heart: hyperdense areas are seen in the heart walls of the atria, right ventricle, and intraventricular septum. (C) Liver: hyperdense round areas are found all over the liver. (D) Adrenal glands: extensive hyperdense areas are found in the bilateral adrenal glands (arrowheads). LA, left atrium; LV, left ventricle; RA, right atrium; RV, right ventricle.

The neonate was treated with surfactant replacement therapy and nitric oxide inhalation for respiratory failure. High-dose catecholamines and hydrocortisone were administered for hypotension and bradycardia. A large amount of blood products was transfused to treat the anemia and coagulopathy. Based on the skin lesions, we suspected an HSV infection and administered acyclovir (10 mg/kg/dose every eight hours). Despite intensive treatment, his condition deteriorated, and he died within 24 hours of life. The postmortem computed tomography revealed an enlarged right lateral ventricle and intraventricular hemorrhage in the brain (Figure 3).

Hyperdense areas consistent with calcification were found in multiple organs: the heart walls, liver, and bilateral adrenal glands. HSV deoxyribonucleic acid was detected in the blood by polymerase chain reaction, and HSV-2 was identified through viral culture from the skin exudates. Although the mother had no symptoms, HSV immunoglobulin M in the mother’s serum was positive with the enzyme immunoassay method (index value 5.09). An autopsy was declined by the parents. Based on the clinical course, the patient was posthumously diagnosed with disseminated intrauterine HSV-2 infection.

## Discussion

To the best of our knowledge, this is the first reported case of intrauterine HSV infection presenting with fetal sinus bradycardia and pericardial effusion. Although we could not obtain histological findings, the patient’s clinical course suggests that he developed HSV myocarditis in utero. Intrauterine HSV infection should be included in the differential diagnosis for fetal or neonatal myocarditis.

Diagnosing fetal myocardial disease is challenging because of the multitude of etiologies and limited diagnostic tools [3]. Frequently reported causes of fetal myocarditis are congenital infection (parvovirus, coxsackievirus, adenovirus, and cytomegalovirus (CMV)) and maternal autoimmune diseases (neonatal lupus) [4]. Although HSV is known to cause myocarditis in children and adults, reports of cardiac complications due to intrauterine HSV infection are limited [5]. We conducted a literature survey for the cases of cardiac complications in intrauterine HSV infection using the PubMed® database with the query of {((congenital) OR (intrauterine) OR (in utero)) AND (herpes)} on July 28, 2025. The search yielded 1,869 records, among which there were nine cases of symptomatic cardiac complications associated with intrauterine HSV infection (Table 1) [6-14].

**Table 1:** Reported and present cases of intrauterine herpes simplex virus infection with cardiac abnormalities. BW, birth weight; CT, computed tomography; GA, gestational age; HSV, herpes simplex virus; IUFD, intrauterine fetal demise; IVS, intraventricular septum; MR, mitral regurgitation; NA, not available; PE, pericardial effusion; RV, right ventricle; TR, tricuspid regurgitation.

Authors (year)	Maternal symptoms	Onset	GA (weeks)	BW (g)	Serotype	Clinical or image findings of the heart	Histological findings of the heart	Other symptoms or histological findings	Outcomes
Von Herzen et al. (1977) [6]	None	At birth	36	2000	2	None	Necrosis in subaortic endocardial area	Skin vesicles, porencephaly, necrosis in the lung, liver, and adrenal glands	Died at 21 days of life
Monif et al. (1985) [7]	Genital HSV-2 infection at 19 weeks of GA	At birth	25	520	2	None	Acute and chronic inflammation of the heart	Skin vesicles and ulcers, meningoencephalitis, and necrosis in the liver and spleen	Died immediately after birth
Beck et al. (1987) [8]	Genital HSV-2 infection at 18 weeks of GA	At birth	25	980	2	Bradycardia	Hemorrhagic necrosis with calcification	Skin ulcer, necrosis in the lung, liver, and adrenal glands	Died immediately after birth
Deshpande et al. (1993) [9]	Premature rupture of membranes	At birth	29	1000	2	Complete atrioventricular block	Necrosis in the heart	Extensive skin exfoliation, pancytopenia, and intraventricular hemorrhage	Died at 4 days of life
Mitra et al. (2004) [10]	Genital HSV-2 infection at 13 weeks of GA	20 weeks of GA	22	NA	2	PE, hyperechogenicity in the RV and IVS	Myocardial necrosis and calcification	Necrotizing pneumonitis	IUFD at 22 weeks of GA
Diguet et al. (2006) [11]	Suspected genital herpes at 11 weeks of GA.	23 weeks of GA	27	680	1	PE	Cardiomegaly	Extensive skin ulcer and splenomegaly	Terminated at 27 weeks of GA
Curtin et al. (2013) [12]	None	20 weeks of GA	22	NA	2	Hyperechogenicity in the RV	Myocardial necrosis, chronic inflammation, and calcification	Cerebral and liver necrosis	Terminated at 22 weeks of GA
Pahlitzsch et al. (2017) [13]	Urinary tract infection	26 weeks of GA	26	920	1 and 2	Hyperechogenicity in the RV, mild TR, and akinesia	Myocardial necrosis and calcification	Extensive skin ulcer, necrosis in the lung, and adrenal glands	IUFD at 26 weeks of GA
Srivastava et al. (2020) [14]	None	23 weeks of GA	23	NA	NA	Fetal bradycardia	Myocardial necrosis	Extensive skin ulcer, liver necrosis	Died immediately after birth
Present case	Placental abruption	26 weeks of GA	28	950	2	PE, sinus bradycardia, MR, TR. Hyperdense areas in the cardiac walls in the postmortem CT	NA	Extensive skin erosion, ventriculomegaly, intraventricular hemorrhage, respiratory failure, coagulopathy, hyperdense areas in the liver and adrenal glands in the CT	Died at 1 day of life

Prenatal abnormalities were detected in five cases: hyperechogenicity of myocardium (n = 3) [10,12,13], pericardial effusion (n = 2) [10,11], and fetal bradycardia (n = 1) [14]. Maternal HSV infection was diagnosed or suspected in four cases [7,8,10,11]. All cases of intrauterine HSV infection with cardiac complications died in utero or in the neonatal period. Myocardial necrosis was found in seven cases in the autopsy [6,8-10,12-14]. Our literature survey revealed that cardiac complications in neonatal HSV infection are rare but are a sign of an unfavorable outcome. Glukhovets et al. performed immunofluorescence testing on the placenta and internal organs of 96 stillbirth fetuses and reported that HSV-1, HSV-2, and CMV were detected in 16 (17%), 19 (20%), and 16 (17%) fetuses, respectively [15]. Among these 52 cases testing positive for these herpes family viruses, myocarditis was observed in 34 (65%) cases. Notably, the pathological changes were predominantly localized in the atrial region, which may cause arrhythmias in the fetus. These findings suggest that a large proportion of intrauterine HSV infection with cardiac involvement may result in abortion or stillbirth, and the actual incidence rate of myocarditis in intrauterine HSV infection may be underreported.

## Conclusions

Intrauterine HSV infection can occur in a pregnant woman without any symptoms, and the number of patients may be underreported due to the high rates of abortion or stillbirth. Clinicians should consider intrauterine HSV infection in cases of unexplained fetal bradycardia or pericardial effusion. Cardiac abnormalities associated with intrauterine HSV infection are a sign of poor outcomes.

## References

[1] James SH, Kimberlin DW (2025). Herpes simplex virus infections. Remington and Klein's Infectious Diseases of the Fetus and Newborn Infant (Ninth Edition).

[2] Fa F, Laup L, Mandelbrot L, Sibiude J, Picone O (2020). Fetal and neonatal abnormalities due to congenital herpes simplex virus infection: a literature review. Prenat Diagn.

[3] Fesslova V, Mongiovì M, Pipitone S, Brankovic J, Villa L (2010). Features and outcomes in utero and after birth of fetuses with myocardial disease. Int J Pediatr.

[4] Donofrio MT, Moon-Grady AJ, Hornberger LK (2014). Diagnosis and treatment of fetal cardiac disease: a scientific statement from the American Heart Association. Circulation.

[5] Bowles NE, Ni J, Kearney DL (2003). Detection of viruses in myocardial tissues by polymerase chain reaction. evidence of adenovirus as a common cause of myocarditis in children and adults. J Am Coll Cardiol.

[6] Von Herzen JL, Benirschke K (1977). Unexpected disseminated herpes simplex infection in a newborn. Obstet Gynecol.

[7] Monif GR, Kellner KR, Donnelly WH Jr (1985). Congenital herpes simplex type II infection. Am J Obstet Gynecol.

[8] Beck R, Park T, Farrington P, Steigman CK, Sennesh JD (1987). Congenital disseminated herpes simplex virus type II infection in a premature infant. Am J Perinatol.

[9] Deshpande PG, Leslie GI, Arnold JD, Bowen JR (1993). Disseminated herpes simplex type II virus infection presenting at birth. Indian Pediatr.

[10] Mitra AG, O'Malley DP, Banks PM, Kelley M (2004). Myocardial calcification in a fetus: a distinctive presentation of in utero herpes simplex virus type II infection. J Ultrasound Med.

[11] Diguet A, Patrier S, Eurin D, Chouchene S, Marpeau L, Laquerrière A, Verspyck E (2006). Prenatal diagnosis of an exceptional intrauterine herpes simplex type 1 infection. Prenat Diagn.

[12] Curtin WM, Menegus MA, Patru MM (2013). Midtrimester fetal herpes simplex-2 diagnosis by serology, culture and quantitative polymerase chain reaction. Fetal Diagn Ther.

[13] Pahlitzsch TM, Helbig ET, Sarioglu N, Hinkson L, von Weizsäcker K, Henrich W (2017). Novel insights in fetal cardiomyopathy due to in utero herpes simplex virus infection. Fetal Diagn Ther.

[14] Srivastava HK, Ellis LT, Miller DC, Duff DJ (2020). Herpes in the heart: a case of widely disseminated intrauterine herpes simplex virus infection involving neonatal myocardium in a 23-week gestationally aged neonate. Case Rep Infect Dis.

[15] Glukhovets BI, Glukhovets NG, Belitchenko NV, Sosunova OA (2016). Immunofluorescence diagnosis of the herpesvirus stillborn infection. Vopr Virusol.

